# In-depth Proteome of the Hypopharyngeal Glands of Honeybee Workers Reveals Highly Activated Protein and Energy Metabolism in Priming the Secretion of Royal Jelly*[Fn FN2]

**DOI:** 10.1074/mcp.RA118.001257

**Published:** 2019-01-07

**Authors:** Han Hu, Gebreamlak Bezabih, Mao Feng, Qiaohong Wei, Xufeng Zhang, Fan Wu, Lifeng Meng, Yu Fang, Bin Han, Chuan Ma, Jianke Li

**Affiliations:** From the ‡ Institute of Apicultural Research/Key Laboratory of Pollinating Insect Biology, Ministry of Agriculture, Chinese Academy of Agricultural Sciences, No. 1 Beigou Xiangshan, Beijing, 100093, China

**Keywords:** Protein Identification*, Protein Synthesis*, Ribosomes*, Molecular biology*, Energy metabolism

## Abstract

Royal jelly (RJ) is a secretion of the hypopharyngeal glands (HGs) of honeybee workers. High royal jelly producing bees (RJBs), a stock of honeybees selected from Italian bees (ITBs), have developed a stronger ability to produce RJ than ITBs. However, the mechanism underpinning the high RJ-producing performance in RJBs is still poorly understood. We have comprehensively characterized and compared the proteome across the life span of worker bees between the ITBs and RJBs. Our data uncover distinct molecular landscapes that regulate the gland ontogeny and activity corresponding with age-specific tasks. Nurse bees (NBs) have a well-developed acini morphology and cytoskeleton of secretory cells in HGs to prime the gland activities of RJ secretion. In RJB NBs, pathways involved in protein synthesis and energy metabolism are functionally induced to cement the enhanced RJ secretion compared with ITBs. In behavior-manipulated RJB NBs, the strongly expressed proteins implicated in protein synthesis and energy metabolism further demonstrate their critical roles in the regulation of RJ secretion. Our findings provide a novel understanding of the mechanism consolidating the high RJ-output in RJBs.

## Abstract (precis)

The hitherto depth proteomes of the hypopharyngeal glands (HGs) across the adult life of two stocks of honeybees with low (ITBs) and high royal jelly production (RJBs) uncover the molecular landscapes of the gland ontogeny and activity to match with gland age-specific tasks. Pathways involved in protein and energy metabolism are induced in HGs of RJB nurse bees to enhance royal jelly (RJ) secretion relative to ITBs. Our finding gains a novel mechanistic insight of the augment RJ-output in RJBs.

## Graphical Abstract

**Figure:**
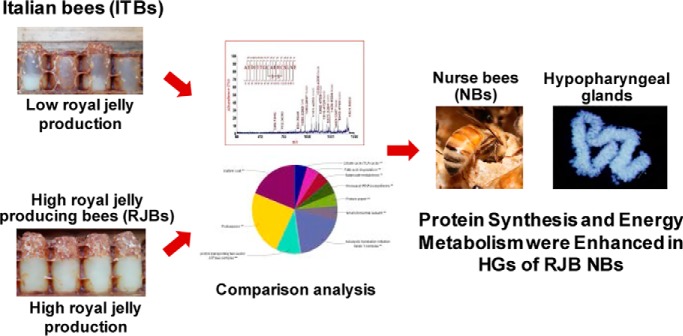

**Highlights**

- In-depth proteome underpins the gland ontogeny and age-specific activity of the HGs.
- The well-developed acini in the HGs of NBs promote the RJ secretary activities.
- The enhanced protein and energy metabolism in the HGs boost the stronger RJ secretion of RJBs.

One of the most important characteristics of honeybees (*Apis mellifera*) in colonies, is the age-dependent role change, known as age polyethism (1, 2). The exocrine hypopharyngeal glands (HGs)^1^ are one of the organs that are subject to age polyethism to achieve their biological mission of secreting larval food: royal jelly (RJ). HGs are coiled in the sides of the head and deliver the proteinaceous secretory product, RJ, via a large collecting duct to the hypopharynx (3–5). Once the worker bees emerge from their cells in the honeycombs, hereafter referred to as newly emerged bees (NEBs, < 24 h after eclosion), the HGs are formed but not fully developed (6). In nurse bees (NBs, 6–15 d after emergence), the HGs develop as an elaborate organ composed of hundreds of acini that are connected and arranged around a collecting duct, and deliver the secreted RJ into the collecting duct, through which RJ runs to the mouthparts (6–9). NBs develop a large size and full morphology of HGs until the onset of foraging (3, 9). In forager bees (FBs, > 15 days after emergence and involved in foraging activity), the size of HGs gradually decreases, and their secretion switches from that of RJ to enzymes for brewing honey and pollen (10–12). The age polyethism of HGs has been well-documented in terms of tasks shifting from nursing to foraging behavior in worker bees (10, 11). However, HGs are also extremely variable in size and morphology with a developmental cycle closely related to the division of labor, depending on the needs of the colony (13–15). Overall, feeding behavior causes HG activation (14, 16, 17).

RJ is a critical food deciding whether fertilized eggs will develop into queen bees or worker bees during the early larval stages (18–22). This is accomplished by modification of the epigenetic state of the genome and gene expression by regulating DNA methylation, thus resulting in genetic morphology development (23). For humans, RJ is widely used in nutraceuticals and cosmetics (20, 24) for its antibacterial and antioxidative properties, and possible anticancer effects (19, 21, 25–27). To increase RJ yields, beekeepers in China have been selecting a stock of high royal jelly producing bees (RJBs) from Italian bees (ITBs, *Apis mellifera* ligustica) since the 1980s. Since then the RJBs have been selected for near 40 years and royal jelly production has been confirmed to be a heritable trait (28). To date, one colony of RJBs can produce more than 10 kg of RJ per year, which is at least 10-fold the production of ITBs (24, 29, 30). Hence, China is the largest producer and exporter of RJ globally, supplying > 90% (∼ 4000 tons/year) RJ to the world market (29, 31). Now, the RJBs are a valuable honeybee resource in China and the world (29–33).

By applying state of the art mass spectrometry (MS), an unprecedented depth of proteome coverage can be attained, and novel knowledge of honeybee biology can be gained. Proteomics is thus becoming the most important tool in delineating honeybee biology at the molecular level (34). For example, the molecular basis of different organs and tissues which allows them to achieve their biological mission have been explained at proteome scales, such as in the brain (33, 35–38), hemolymph (32), embryo (39, 40), and antennae in *Varroa* resistance (41, 42). Moreover, during the past few years, our research group has conducted a wide spectrum of studies to reveal the molecular basis that supports the enhanced RJ production by RJBs. For instance, an enhanced level of neuropeptides is implicated in regulating water homeostasis, brood pheromone recognition, foraging capacity, and pollen collection in RJBs to regulate the behavior of RJ secretion (35). The activities of phosphatidylinositol signaling and arachidonic acid metabolism in RJB NBs are elevated to increase olfactory sensation in response to larval pheromone stimulation (33). The selective enhancement of the mandibular glands of RJBs on the activity of pathways related to lipid synthesis to maintain a proper proportion of 10-hydroxy-2-decenoic acid (10-HAD), a vital fatty acid in RJ for larval nutrition, fits the increased RJ production (30). Furthermore, RJBs have reshaped their hemolymph proteome by providing surplus protein and energy molecules to prime augmented RJ outputs (32).

Despite the importance of HGs for RJ production, only one study has been done that compares the proteome differences between ITBs and RJBs, by using two-dimensional electrophoresis-based (2-DE) proteomics (9). Because of technical limitations, only a small fraction of proteins (116 proteins) were identified in HGs, thus more in-depth understanding of the molecular basis of high RJ production in RJBs is required. Therefore, the aim of this work was to investigate and compare the age-specific HG proteome of NEBs, NBs, and FBs between ITBs and RJBs by using cutting-edge high-resolution MS-based proteomics. The functions of proteins in the critical pathways that are implicated in the secretory activity of RJ in HGs had been validated by two behavioral experiments, through which the worker bees were manipulated to extended nursing periods or the FBs were forced to revert into NBs. This work provides a novel insight into the mechanism with which the HGs achieve enhanced production of the valuable bee-product, RJ.

## EXPERIMENTAL PROCEDURES

### 

#### 

##### Experimental Design and Statistical Rationale

The colonies used for sampling the HGs of ITBs and RJBs (*Apis mellifera ligustica*) were kept at the Institute of Apicultural Research, Chinese Academy of Agricultural Sciences, Beijing. The queens of the ITBs were from Bologna, Italy, and the queens of RJBs were from Zhejiang Province, China, where the RJBs were selected and maintained in a breeding station. All colonies were managed with almost identical population structure, brood pattern, and food during the nectar flow of chaste berry (*Vitexnegundo* L.) in June.

**Fig. 1.**
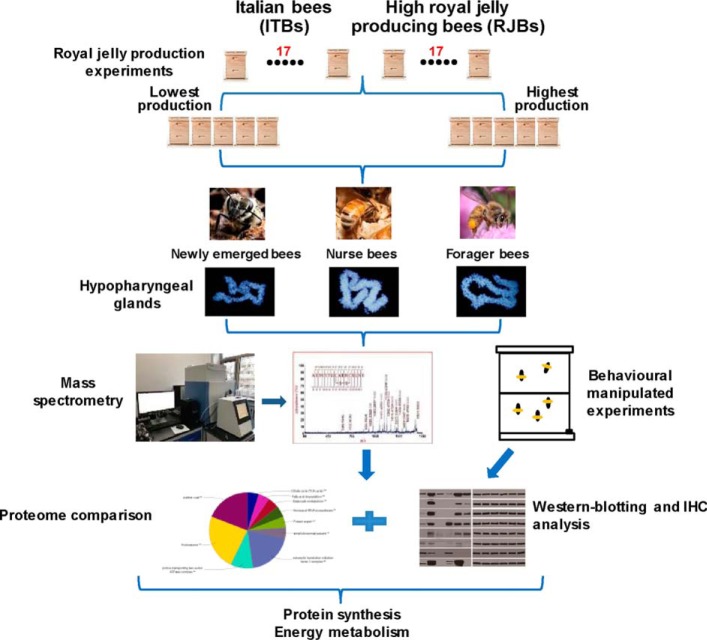
**The framework to study the enhanced functions in hypopharyngeal glands (HGs) of high royal jelly producing bees to boost royal jelly (RJ) production.** We investigated the RJ production from 34 colonies of both Italian bees (ITBs) and high royal jelly producing bees (RJBs) (17 colonies of each line). Following the RJ production experiments, HG samples were collected from 10 typical colonies (5 colonies of each line) and analyzed by proteome comparison and biochemical verifications between ITBs and RJBs across various developmental stages of worker bees. We managed two social manipulation experiments to assess performance of a behavior phenotype in sampling for reversed nurse bees and prolonged nurse bees. The procedures of two social manipulation experiments were performed as in supplemental Fig. S1, and for the details of the experimental methods see Experimental Procedures.

The workflow of the investigation and comparison of the HG proteome during 3 stages of worker bees between ITBs and RJBs is shown in Fig. 1. A total of 34 colonies (17 colonies from each strain) received similar management practices to attain comparable colony size for measuring RJ production. The collection method of RJ was performed as previously described, with minor modifications (9, 22). The young worker larvae (> 24 h old) were grafted into to the cell cups, the accepted larvae were fed royal jelly by nurse bees, and the unaccepted larvae (if there were) were removed by worker bees. After around 72 h of larval grafting, the accepted queen cell numbers were counted, and RJ was collected from each colony. The rate of queen cell acceptance is the accepted cell number divided by the total grafted cell number, 128. RJ of each colony was sampled five times, and the samples were weighed with a digital scale (Mettler Toledo, Columbus, OH; accurate to 0.001 g).

After the RJ production survey, five colonies of RJBs with the highest RJ outputs and five colonies of ITBs with the lowest production were selected for HG sampling. Honeybees from five colonies per time point were collected and pooled as one sample. For each time point (at NEBs, NBs, and FBs), at least 100 bees were sampled from each colony. And triplicates were produced in each sample for LC-MS/MS analysis. The expressional profile of regulated proteins between different samples in all proteomics was created by the PEAKS software using an un-centered Pearson correlation and average linkage. All proteomics and western-blotting (WB) data were evaluated with Student's *t* test, and a *p* value of < 0.05 was significant. Graph was represented by the mean and S.D.

##### Sampling HGs of Worker Honeybees

Parts of NEBs (< 24 h) were sampled when they emerged from sealed brood in an incubator, and others were then marked on their thoraxes with paint dots and returned to the colonies for further development. During the following 8–10 days, the marked bees were collected as NBs when they were observed to extend their head into the cells containing the young larvae. The FBs, carrying pollen loads, were collected at the hive entrance after 15 days of emergence. The HGs were dissected in cold PBS containing protease inhibitors (Roche; Indianapolis) with the help of a binocular microscope, and the samples were then pooled based on age and bee colony and stored at −80 °C for later analysis.

##### Honeybee Social Manipulation to Get Reversed NBs and Prolonged NBs

To exclude the effect of age on the expression of candidate proteins and validate that the biological functions of the proteins are related to secretory behavior of RJ rather than age regulation, two social manipulation experiments to regulate behavioral biology of honeybees were performed twice, in order to reveal the proteins' functions in the regulation of RJ secretory biology. The two experimental strategies were performed as shown in supplemental Fig. S1.

The purpose of experiment 1 (supplemental Fig. S1*A*) was to collect normal NBs, FBs, and reversed nurse bees (RNs) according to the previously described protocol (43–45). In brief, control forager bees (CFs) were the regular worker bees that were observed showing foraging behavior 15 days after eclosion. RNs were the FBs 15 days after eclosion that exhibited nurse-like behavior again in the brood cells after manual manipulation, *i.e.* observed with head extension in brood cells. Regular NBs were the bees that showed nursing behavior 8–10 days after eclosion. To get these three types of bee samples, the first step was to organize a queen-right host colony with about 6,000 unmarked workers of diverse ages. Thereafter, a total of > 10,000 NEBs from three RJB colonies (about 500 NEBs each) were labeled on the thorax and put in the host colony for development of upcoming normal hive activities, such as nursing and foraging. After solid foraging behavior was established, the marked FBs were color-labeled on their abdomen with different colors to track their life history. The hive entrance observations of the marked FBs returning from foraging were logged daily during the most active time of day (8:00 a.m. to 10:00 a.m.) until day 25.

To obtain RNs, in the early morning of day 26 (∼6:00 a.m.), a new hive (keeping colony) was manipulated inside the original host colony. The keeping hive existed of an upper and bottom box separated by a 3 mm wire mesh screen to prevent bees from crossing over between the two boxes. The entrance of the bottom box was maintained in the same direction as the former entryway of the host hive, however, the entrance of the upper box was transferred back (180°) from the direction of the entryway of the bottom box. First, the queen of the host colony was moved into the bottom box of the keeping hive, in which two frames with larvae and two frames with food (honey and pollen) but without workers were placed. Second, all bees and frames without queen in the host colony were moved into the upper box. The keeping hive remained on the site throughout the peak foraging hours of the day. Once experienced foragers in the upper box exited to collect food outside of the hive, they used their pre-acquired memory of the hive site and returned from the entryway of the original host colony to contribute to the worker bees of the keeping colony. At 12:00 am, the entrance of the upper box was closed and then moved to a location several kilometers away. Afterward, a queen, larvae, food, and a population of worker bees (consisting of experienced foragers) were engaged in the colony activity of the keeping hive. Applying above colony manipulation of social context, foragers will revert to nursing activities (17, 46). RNs were then collected during 4–8 days after the setup of the keeping hive. The labeled control FBs were collected when they returned from flights with pollen during the peak foraging hours from the host colony.

Experiment 2 (supplemental Fig. S1*B*) was performed to manipulate honeybee behavior by ethyl oleate (EO), a chemical inhibitory factor presented in high concentrations in the honey crop, which can be transmitted via trophallaxis (a form of food exchange as a prominent communication channel in insect societies), thus delaying the age of onset of adult FBs. To prolong nursing behavior, EO treatments of colonies were performed following a previously described protocol (47). Bees were exposed to EO by ingesting sugar candy containing EO (30% honey, 70% powdered sugar). One gram of EO-containing candy was replaced daily and the dose of EO was 2.1 mg/g of sugar candy. The effect of EO was tested in the field with two cohort colonies containing bees (*n* = 500 per host) from three host colonies with different ages to roughly simulate the normal range of worker ages within a colony, as previously described (17).

To control genotypic variation, colonies in the trial were all built from the same RJB colony and were made with similar size, demography, honeycomb number, brood, and location in the field. All colonies with a similar number of bees were placed in small beehives that contained two combs (one with honey, one without). Two colonies were treated with sugar candy containing the dose of EO and two were given sugar candy alone as control (CF-EO).

To sample worker bees with a known age, NEBs were marked on the thorax with a separate color for each cohort. The NBs and FBs were sampled as described above. To manage the worker behavioral maturation, honeybee primer pheromones, queen mandibular pheromone (QMP), and brood pheromone (BP) were artificially controlled. Instead of a live queen, each colony was given a plastic strip (Bee Boost, PheroTech, Vancouver, Canada) containing the five-component QMP blend that released one queen-equivalent per day (47–49). Because no brood was produced, colonies had no exposure to BP. The pheromone strips were replaced every 2 weeks.

The effect of the EO treatment on the rate of behavioral maturation was quantified by determining the mean age at which the first 50 bees from each treatment-initiated foraging. Regular observation was started several days before any colonies exhibited foraging. The hive entrance was observed daily during the most active time of day (8:00 a.m. to 4:00 p.m.) in four 30-min observation periods per colony. A plastic screen was placed in front of the hive entrance for forager bees to enter the hive one by one, thus slowing down the speed of entrance. When the marked forager bees were observed to go through the screen, they were given an additional paint marking by automation and their ages and behavior were recorded. These were considered normal FBs and not collected. After 3 days from the first day, it was observed that foraging was initiated, and the marked focal worker bees without the paint marking were collected as prolonged nurse bees (PNs).

More than 100 worker bees were collected in each group of both experiments. For all bees, individuals from both experiments were collected and directly stored at 4 °C until the HGs were dissected (< 24 h). The samples of HGs were immediately transferred into liquid hydrogen and stored at −80 °C until use.

##### Protein Extraction and Digestion

Protein extraction was performed as previously described (35, 50). Unless the source is specified, all chemicals were purchased from Sigma-Aldrich (St. Louis, MO). All the reagents were of analytical or HPLC grade. In short, prior to protein extraction the HG tissue was homogenized in liquid hydrogen with a pestle. The HGs were then mixed with a lysis buffer containing 8 m urea, 2 m thiourea, 4% 3-[(3-cholamidopropyl) dimethylammonio]-1-propanesulfonate, 20 mm Trisbase, 30 mm dithiothreitol (DTT), and protease inhibitors (Roche, Mannheim, Germany) in ice for 30 min. Afterward, the sample was further centrifuged at 15,000 g for 20 min at 10 °C to remove the insoluble fractions. Three volumes of ice-cold acetone were added to the recovered supernatant at −20 °C for 4 h to precipitate the proteins. Subsequently, the protein pellets were centrifuged at 8,000 g at 10 °C for 20 min. The supernatant was discarded, followed by extraction of the protein pellet at room temperature (RT). The recovered proteins were re-suspended in 100–150 μl of 5 m urea, and protein concentration was quantified by a Bradford assay after diluting 50 times. Of each sample, 200 μg of proteins were used by adding four volumes of 40 mm NH_4_HCO_3_, mixing with DTT (final concentration 10 mm) for 1 h, and then alkylating with iodoacetamide (final concentration 50 mm) for 1 h in the dark. The surplus iodoacetamide was quenched by DTT (final concentration 30 mm). To digest protein into peptides, sequencing grade modified trypsin (Promega, Medison) was used (enzyme/protein ratio of 1:100 (W/W)) at 37 °C for 14 h. The enzymatic digestion was stopped by adding 1 μl of formic acid to the solution. The digested peptide samples were desalted by a C18 column (Agilent Technologies Inc., Santa Clara, CA). The eluted peptide solution was collected and extracted using a SpeedVac system (RVC 2–18, Marin Christ, Osterod, Germany) and stored at −80 °C for subsequent LC-MS/MS analysis.

##### LC-MS/MS Analysis

The digested peptide samples were re-dissolved in 50 μl of 0.1% FA. Three replicates of each sample were run using a Q-Exactive mass spectrometer (Thermo Fisher Scientific) and coupled to the EASY-nLC 1000 system using a nano electrospray ion source (Thermo Fisher Scientific). To enrich the peptide samples, they were first loaded onto a 2 cm long trap column (75 μm inner diameter fused silica containing 3 μm Aqua C18 beads, Thermo Fisher Scientific) for 2 min in buffer A (0.1% acetic acid) at a flow rate of 10 μl/min. Secondly, the peptides were separated by an analytical column (15 cm long, 50 μm inner diameter fused silica column filing with 2 μm Aqua C18 beads, Thermo Fisher Scientific) using a 120 min gradient. Peptides were gradient eluted for 110 min with a linear gradient from 8% to 30% acetonitrile at a flow rate of 300 nL/min. The eluting peptides from the analytical column were directly infused into a Q-Exactive mass spectrometer (Thermo Fisher Scientific) via electrospray ionization. The settings for a data-dependent mode to collect the MS and MS/MS data were as follows: one full scan (resolution 70,000 at 400 *m*/*z*; 350 to 1600 *m*/*z*) followed by top 20 MS/MS scans using higher-energy collisional dissociation in the linear ion trap mass spectrometer (resolution: 15,000, isolation window: 2 *m*/*z*, normalized collision energy: 28) using dynamic exclusion (charge exclusion: unassigned 1, >8; peptide match: preferred; exclude isotopes: on; dynamic exclusion: 30 s).

##### Identification and Abundance Level Quantification of Proteins

The MS/MS data in RAW were retrieved using Xcalibur (version 3.0, Thermo Fisher Scientific) and searched using in-house PEAKS software (version 8.5, Bioinformatics Solutions Inc., CAN). A database containing protein sequences of *Apis mellifera* including common contaminants was downloaded from NCBI and used, totaling to 22,757 entries (downloaded 18 April, 2017). The parameters of the search database were as follows: trypsin; maximum missed cleavage: 2; precursor ion and MS/MS tolerances: 15 ppm and 0.05 Da; a fixed modification: carba midomethyl (C, +57.02); and a variable modification: oxidation (M, +15.99). The fusion-decoy database search strategy with threshold FDR ≤ 1% was used to control the false discovery rate (FDR) at both the protein and peptide levels (51). A protein was considered as identified only if it had at least two unique peptides. The MS proteomic data have been deposited to the ProteomeXchange Consortium (http://Proteomecentral.Proteomexchange.org) via the PRIDE partner repository with the data set identifier PXD010898.

To quantify the relative level of protein abundance in the HGs of both ITBs and RJBs, three replications of each sample were performed in the quantification module of PEAKS software (version 8.5) via a label-free strategy. Feature detection was performed separately on each sample using the expectation-maximization algorithm. Using the high-performance retention time alignment algorithms, the features of the same peptide from three replicates of each sample were reliably aligned (51). Normalization was done by dividing each matrix by a factor of the samples obtained as follows: the total ion current (TIC) of the individual sample/the TIC of the reference sample. Quantification of protein abundance levels in the HGs in all samples of both bee lines was done using the sum of the three highest ion peak intensities of the tryptic peptides. Furthermore, the protein abundance levels were compared between NEs, NBs and FBs of both bee stocks and between each time point of NEs, NBs, and FBs of both bee lines.

##### GO Term Enrichment Analysis

To understand the biological implications of the identified proteins in the HGs of worker honeybees, identifiers of protein GI numbers were used as an input for GO term enrichment (functional classes and pathway) using ClueGOv2.3.2, a Cytoscape plug-in (http://www.ici.upmc.fr/cluego/) (Bindea, 2009). The number of proteins identified from the samples was compared with the number of functionally GO annotated proteins in the entire honeybee (*Apis mellifera.* L) genome for enrichment analysis. A right-sided hyper-geometric test was used to report the significantly enriched functional GO categories and pathways in biological processes. A Bonferroni test was used to account for FDR. The GO terms and pathways were considered as significantly enriched only when the corrected *p* value was < 0.05. The kappa score (0.4) in ClueGo was mandatory to link the nodes in the functional networks.

##### WB and Immunohistochemistry (IHC) Assay for Protein Expression

To confirm the expression variations of proteins implicated in RJ secretion in HG samples in different phenotypes, the WB and IHC were performed as previously described (10, 36, 52). And the proteins remarkable changed in their abundance levels or significantly enriched in the biological processes or pathways were selected for validation. Polyclonal rabbit antibodies against 60S ribosomal proteins RpL28, RpL26, and 40S ribosomal protein S4 (RpS4), and the monoclonal mouse antibody against major royal jelly protein 1 (MRJP1) were developed (Genecreate Biological Engineering, Wuhan, Hubei, China). Monoclonal mouse antibodies against (RpL4), 40S ribosomal protein S3a (RpS3A), hexamerin 110 (Hex110), Lamin Dm0 (Lam), Malate dehydrogenase (MDH), UTP-glucose-1-phosphate uridylyltransferase (UGP2), citrate synthase (gltA), and β-Actin were developed (Abmart. Shanghai, China). The specificity of the above antibodies was then tested by Genecreate and ABmart. The second antibodies were purchased from Sigma. For WB assay, the primary antibodies were diluted at a ratio of 1:1000–3000, and the secondary antibodies were diluted at a ratio of 1:5000.

About 40 μg of total proteins of each sample were separated by stacking (5%) and separating (10%) on sodium dodecyl sulfate-polyacrylamide gel electrophoresis (SDS-PAGE) under denaturing conditions at 80 v for 0.5 h and 120 V for 1.5 h, and then transferred to a polyvinylidene fluoride membrane (Merck Millipore Ltd, Darmstadt, Germany) under 220 mA constant current for 1.5–2 h. The membrane was blocked for 1 h with western blocking buffer (Applygen Technologies Inc, Beijing, China) at room temperature. Following washing with TBST (Applygen Technologies Inc, Beijing, China) 3 × 10 min, primary antibodies were added and incubated at 4 °C overnight. The membrane was then washed with TBST 3 × 10 min and incubated with the secondary antibody at RT for 1 h. The enhanced chemiluminescent Novex ECL substrate (Thermo Fisher Scientific) enabled immunodetection of horseradish peroxidase-conjugated secondary antibody using films.

For IHC assay, bees were anesthetized at 4 °C, and the HGs were dissected in a solution of cold PBS (phosphate-buffer saline, 6.7 mm) with a protease inhibitor mixture. The samples were then fixed in 6.7 mm PBS containing 4% paraformaldehyde at RT for at least 48 h, and the tissues were dehydrated in ethanol baths in the following order: 70% Ethanol 20 min; 95% Ethanol 2 × 20 min; 100% Ethanol 2 × 20 min. The tissues were embedded with melted paraffin and sectioned in 4–10 μm thickness onto clean glass slides by a microtome (Leica RM2235, Nussloch, Germany). After treatment with blocking solution (1% anti-goat IgG and 0.5% Triton X-100 in blocking solution for 50 min at RT), the slices were incubated in primary antibodies (diluted by blocking solution, 1:150) overnight at 4 °C. After washing 3 × 15 min, specimens were stained with AlexaFluor 488 phalloidin (Invitrogen, Eugene, OR) or Cy3 (Biotium Inc., Fremont, CA) for 1 h, and then with 4′, 6-diamidino-2-phenylindole (DAPI, Beyotime Biotech, Hangzhou, China) for 5 min. Afterward, a drop of DAPI Fluoromount-GTM was covered to quench fluorescence, color intensities were then measured in the MicroPublisher 5 RTV (QImaging, Surrey, Canada).

## RESULTS

### 

#### 

##### RJ Production

**Fig. 2.**
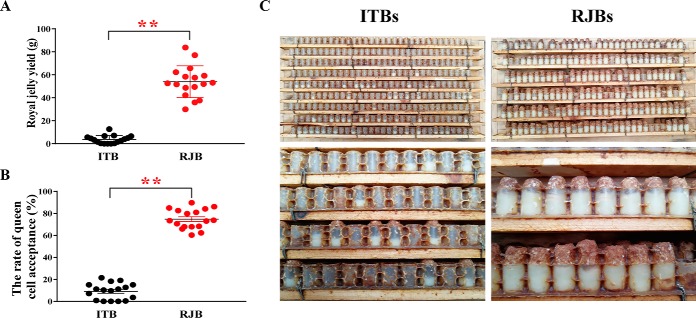
**Royal jelly (RJ) production.** *A*, The average weights of RJ collection (shows as mean ± S.D., *n* = 17) of Italian bees (ITBs) and high royal jelly producing bees (RJBs) from 34 colonies (17 colonies of each line). *B*, The average queen cell acceptance rate (shows as mean ± S.D., *n* = 17) of ITBs and RJBs from 34 colonies (17 colonies of each line) over five collection time points. Every dot is an average value of one colony for five times. Asterisks indicate the statistically significant differences between ITBs and RJBs (*p* < 0.01). *C*, A royal jelly frame with wax caps on the queen cell cups from both ITBs and RJBs collected around 72 h after larval grafting. The acceptance numbers of queen cell cups of ITBs is fewer than those of RJBs, and the content in one cup of ITBs is about a third of that of RJBs.

The average RJ productions of ITBs and RJBs were 3.7 ± 0.84 g and 54.0 ± 3.4 g, respectively (Fig. 2*A*). With a difference of over 10-fold, the RJ production of RJB was significantly higher than that of ITBs. The average rate of queen cell acceptance of RJBs (75%) is remarkably higher than that of ITBs (10%) (Fig. 2*B*).

##### Quality Assessment of Proteomics Data

To achieve an in-depth mechanistic understanding of the development and functionality of HGs and the difference between two honeybee stocks with different phenotypes of RJ production, the proteome of the HGs across three time points of worker bees was analyzed and compared between ITBs and RJBs. Overall, 2375 protein groups were identified in both honeybee strains, in which 435 secretory proteins (supplemental Table S1) existing in the HGs were removed from the identified proteins including the previously reported 98 putative RJ proteins (50, 53–56). Of the total 2375 protein groups, 2097 (supplemental Table S2) and 2084 (supplemental Table S3) protein groups were identified in ITBs and RJBs over three time points, respectively, of which 1806 protein groups were shared by both honeybee strains (Table I).

**Table I:** The identified protein group numbers of hypopharyngeal glands in three stages of both Italian bees (ITBs) and high royal jelly producing bees (RJBs)

Protein groups	NEBs^a^	NBs^b^	FBs^c^	Total
ITBs	1983	1257	1455	2097
RJBs	1814	1387	1421	2084
Total	2090	1501	1706	2375
Differentially expressed	47	200	338	—
Upregulated in ITBs	22	14	177	—
Upregulated in RJBs	25	186	161	—

^a^NEBs, Newly emerged bees;

^b^NBs, nurse bees;

^c^FBs, forager bees.

**Fig. 3.**
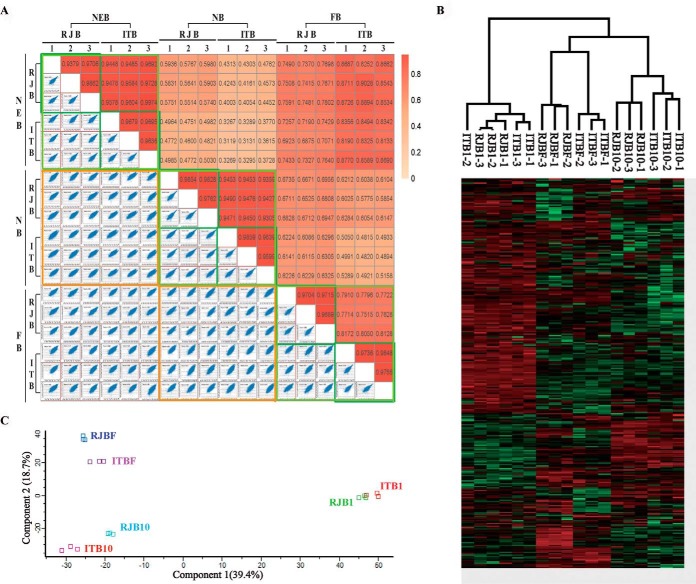
**Quality assessment of proteome data by label-free quantification (LFQ).** *A*, Assessment of MS data quality. The matrix of 18 correlation plots covering 3 stages during the development of hypopharyngeal glands (HGs) in both Italian bees (ITBs) and high royal jelly producing bees (RJBs) reveals very high correlations between LFQ intensities in triplicates (Pearson correlation coefficient 0.94–0.98 between replicates, cycled by dark green squares), demonstrating the technical reproducibility. The color code follows the indicated values of the correlation coefficient. *B*, Principal component analysis (PCA). The proteome of NEB HGs in triplicates segregated into organs based on component 1 and component 2, which account for 39.4% and 18.1% of variability, respectively. *C*, Hierarchical clustering analysis of identified proteins in each sample.

To evaluate the quality of the age-dependent proteome data of HGs, 18 MS/MS raw files of NEBs, NBs, and FBs in both ITBs and RJBs were analyzed with the Pearson's correlation coefficient, principal component analysis (PCA), and clustering analysis (Fig. 3). The Pearson correlation coefficients of all replicates for each of the samples were > 0.94 (marked by dark green squares), and the ones between the same ages of the two bee stocks also had a high correlation (marked by light green squares), especially those of NEBs (> 0.94) and NBs (> 0.93) (Fig. 3*A*). However, the correlations between different stages were much lower, with a correlation between NEBs and NBs of less than 0.6, and between NBs and FBs ranging between 0.5–0.7 (marked by orange squares). In the PCA, NEBs, NBs, and FBs were separately clustered, and the cluster of NEBs was far from NBs and FBs (Fig. 3*B*). Moreover, the proteome profiling of all quantified proteins across the six samples of each stage were clustered into three independent branches, *i.e.* NEBs, NBs, or FBs of ITBs and RJBs were clustered as independent clusters (Fig. 3*C*). This data demonstrates a high reproducibility of technical replicates in the mass spectrometry measurement.

##### Proteome Comparisons Reveal the Gland Ontogeny and Age-specific Task Activity

Qualitatively, in ITBs, of the 2097 protein groups identified (supplemental Table S2), 1983, 1257, and 1455 were found in the NEBs, NBs, and FBs, respectively (Table I, supplemental Table S4, S5 and S6). In RJBs, of the 2084 protein groups identified (supplemental Table S3), 1814, 1387 and 1421 protein groups were found in the NEBs, NBs and FBs respectively (Table I, supplemental Table S8, S9, and S10). The qualitative comparisons of ITBs or RJBs across 3 time points were shown in supplemental Fig. S2 and supplemental Table S7, and supplemental Fig. S3 and supplemental Table S11, respectively.

**Fig. 4.**
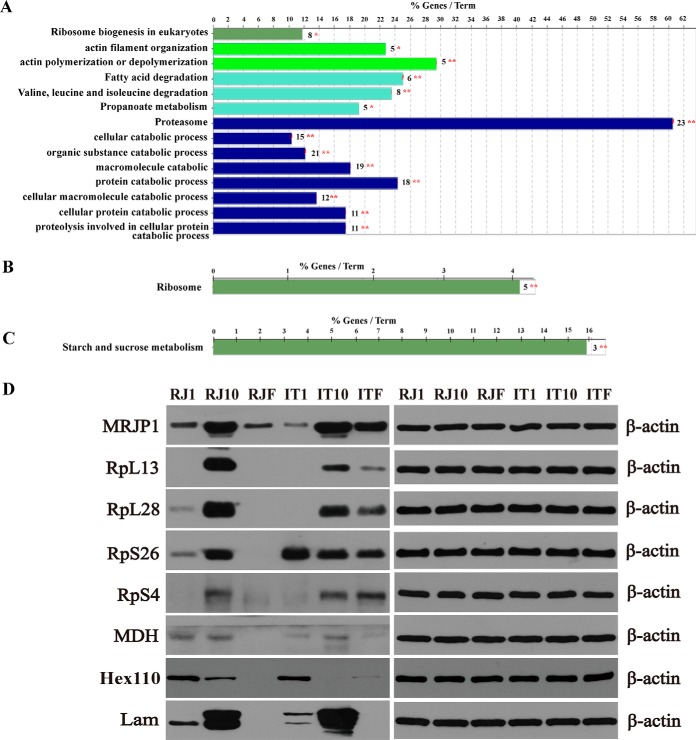
**Qualitative proteome comparisons of age-specific hypopharyngeal glands (HGs) in both Italian bees (ITBs) and high royal jelly producing bees (RJBs).** *A*, *B*, and *C*, The functional classes and pathways enriched by upregulated proteins (fold change ≥2 and *p* < 0.05) in newly emerged bees (NEBs), nurse bees (NBs), and forager bees (FBs), respectively. % genes/Term stands for the proportion of genes enriched in corresponding functional groups. The bars with the same color represent the same functional groups they belong to. The numbers stand for the genes enriched to the corresponding functional groups. For details of the enrichment analysis results see supplemental Table S15. *, *p* < 0.05; **, *p* < 0.01. *D*, The validated abundance level change of proteins related to protein biosynthesis and energy metabolism in NEBs, NBs, and FBs by western-blotting assay. Protein β-actin is used as a loading control. RJ1, NEBs of RJBs; RJ10, NBs of RJBs; RJF, FBs of RJBs; IT1, NEBs of ITBs; IT10, NBs of ITBs; ITF, FBs of ITBs.

Quantitatively, among the 2097 identified protein groups over the three ages of HGs in ITBs, 737 proteins were significantly regulated, of which 609, 19, and 109 protein groups were upregulated in the NEBs, NBs, and FBs, respectively (Table I and supplemental Table S12). Regarding the 2084 identified protein groups over three ages of HGs in RJBs, the expression levels of 450 protein groups were significantly altered, of which 362, 35, and 53 protein groups were upregulated in the NEBs, NBs, and FBs, respectively (Table I and supplemental Table S13). Overall, among the 609 protein groups regulated in ITBs and 450 in RJBs, the abundance levels of 324 protein groups were significantly changed during HG development in both (Table I and supplemental Table S14). The highly abundant proteins in NEBs were uniquely enriched in actin polymerization or depolymerization, and 3 pathways: valine, leucine and isoleucine degradation, ribosome biogenesis in eukaryotes, and proteasome (Fig. 4*A*, supplemental Table S15). Only ribosome pathway was enriched in NBs (Fig. 4*B*, supplemental Table S15), and only the pathway of starch and sucrose metabolism was enriched in FBs (Fig. 4*C*, supplemental Table S15).

Seven proteins related to energy metabolism and protein synthesis across three ages of HGs in both bee stocks were selected for a WB test. The protein abundance levels were remarkably varied over the three stages and the trend in both strains were generally consistent with the proteome data (Fig. 4*D*). The cytoplasmic and nucleolar protein Hex110 was highly abundant in NEBs. The proteins MRJP1, MDH, Lam, RpL13, and RpL28 were highly abundant in NBs.

##### Well-developed Acini and Cytoskeletal Organization of HGs Promote the RJ Secretory Performance in NBs of Both Stocks

**Fig. 5.**
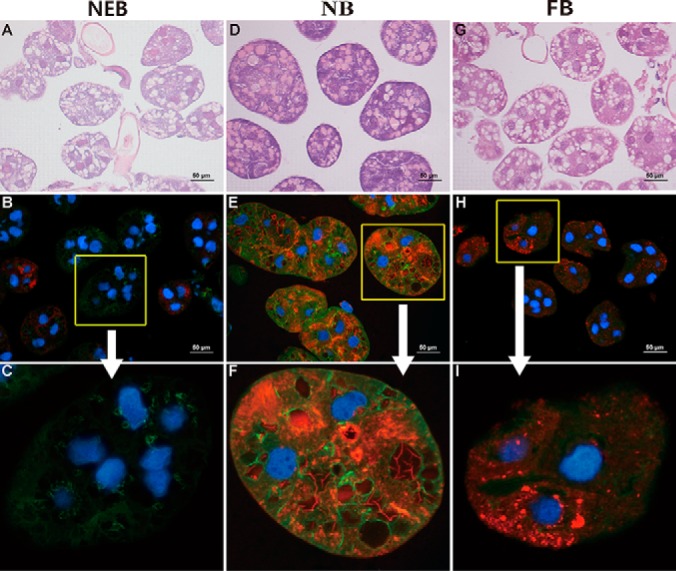
**Morphology of acini in hypopharyngeal glands (HGs).** *A*, *D*, and *G*, The morphology of age-dependent acini stained with hematoxylin-eosin. Hematoxylin colored nuclei cells bluish violet, and eosin bound to the amino acids/proteins in the cytoplasm and stained pink. *B*, *E*, and *H*, The merged images of the immunochemistry staining of the major royal jelly protein 1 (MRJP1) and β-actin in HGs. MRJP1 shows red fluorescence and β-actin shows green fluorescence. *C*, *F*, and *I*, The amplified photographs of circled part in section *B*, *E*, and *H*, respectively. NEB, newly emerged bees; NB, nurse bees; FB, forager bees. Blue is nuclei stained by 4′, 6-diamidino-2-phenylindole. The red fluorescence is stained with Cy3-conjugated antibodies. The green fluorescence is stained by AlexaFluor 488 phalloidin-conjugated antibodies. Scale bars represent 50 μm.

The morphology and HG size were visualized by DNA-binding 4′, 6-diamidino-2-phenylindole (DAPI) and hematoxylin-eosin. MRJP1 was used to evaluate the secretory activity of HG with age development. To localize the key protein expressions related to gland functions, actin filament was visualized using IHC to observe the cytoskeleton structure change of the tubule membrane, which provides a stabilizing framework to the canalicular membrane system during high exocytic activity. The alterations of morphology and size were matched with the HG activity and physiology. In NEBs, the HG acinus was observed as a cavity and less secretory content MRJP1 was available (Fig. 5*A*, 5*B*, and 5*C*). In NBs, the HGs were well-developed, containing the voluminous and numerous acini of ovoid shape linked via bundles of ductules to the collecting duct that extended over the entire length of the gland (Fig. 5*D*). Moreover, MRJP1 was highly abundant in the entire acini (Fig. 5*E* and 5*F*). In FBs, the HGs were decreased in size (Fig. 5*G*) and the secretory ability was caused/supported by the less abundant secretory protein MRJP1 (Fig. 5*H* and 5*I*).

##### The Enhanced Functions of Protein Synthesis and Energy Metabolism in the HGs of RJBs Are Crucial to Cement the Elevated RJ Production

To understand the proteome differences of HGs between ITBs and RJBs at specific ages, the proteomes of each time point were compared. The qualitative and quantitative comparisons of HG proteomes in NEBs or FBs had been presented in supplemental Fig. S4 and supplemental Fig. S5, respectively. The proteomes of HGs displayed an age-dependent program in both strains to consolidate the transformation of behavioral missions. However, a profound difference in the proteome profile was observed in the nursing stage.

**Fig. 6.**
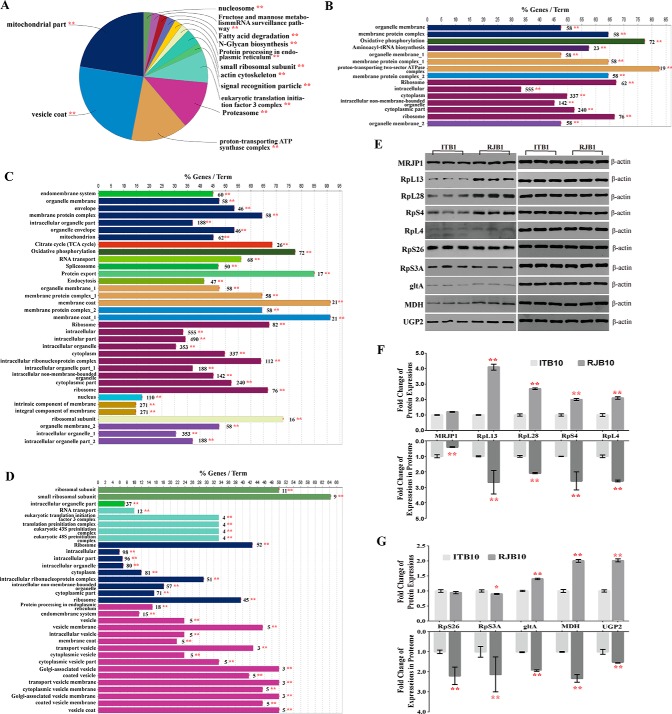
**Comparisons of hypopharyngeal gland (HG) proteins in nurse bees (NBs) between Italian bees (ITBs) and high royal jelly producing bees (RJBs).** *A*, *B*, and *C*, A qualitative comparison of identified HG proteins in NBs between ITBs and RJBs using ClueGO; The proteins identified in ITBs and RJBs are analyzed by ClueGO to compare the functional classes and pathways specifically enriched by two data sets. a, Pie chart overview of the significantly enriched functional classes and pathways shared by both ITB and RJB NBs; b and c, The unique functional classes and pathways significantly enriched by NBs of ITBs and RJBs, respectively. The details of the enrichment analysis results see supplemental Table S19. *D*, The enriched functional classes and pathways of quantitative comparison by upregulated proteins in NBs of RJBs relative to ITBs (fold change ≥ 2 and *p* < 0.05). The details of the enrichment analysis results see supplemental Table S21. % genes/Term stands for the proportion of genes enriched in corresponding functional groups. The bars with the same color present belong to the same functional groups. The numbers stand for the genes enriched to the corresponding functional groups. *E*, The different expressions of proteins in NBs between ITBs and RJBs tested by a western-blotting assay. Protein β-actin is used as a loading control. IT10, NBs of ITBs; RJ10, NBs of RJBs. *F* and *G*, The relative expression value of selected proteins; upside, the relative fold changes of selected protein expressions of the RJBs compared with ITBs tested by WB; downside, the fold changes of key proteins of the RJBs compared with ITBs calculated by label free quantification. Graph was the mean and S.D. *, *p* < 0.05; **, *p* < 0.01.

Of the 1501 identified protein groups in NBs, 1257 and 1387 were found in the HGs of ITBs and RJBs, respectively (supplemental Table S5 and supplemental Table S9). There were 8 GO terms and 5 pathways commonly enriched in both honeybee lines. Among them, the strongly enriched functional groups were: mitochondrial part, vesicle coat, fatty acid degradation, protein processing in ER, actin cytoskeleton, proteasome, and fructose and mannose metabolism (Fig. 6*A*, and supplemental Table S19). In ITBs, organelle membrane and aminoacyl-tRNA biosynthesis were exclusively enriched, relative to RJBs (Fig. 6*B*, supplemental Table S19). In RJBs, 6 GO terms and 5 pathways were uniquely enriched comparing to that of the ITBs, of which ribosomal subunit, TCA cycle, spliceosome, and protein export were strongly enriched (Fig. 6*C*, and supplemental Table S19).

Among the 1501 protein groups identified in the NBs, 200 proteins altered their expression levels, of which 14 and 186 proteins were upregulated in the ITBs and RJBs, respectively (supplemental Table S20). The highly abundant proteins in the RJBs were enriched in functional groups implicated in protein biosynthesis, such as eukaryotic translation initiation factor 3 complexes, and ribosome and protein processing in ER (Fig. 6*D* and supplemental Table S21). However, there were no GO terms enriched in the ITBs by the upregulated proteins.

Secretion of larval food (RJ) is mainly achieved by the HG functions of NBs, and for RJBs the RJ production was over 10-fold higher than ITBs. To validate the driving force of the stronger RJ secretory activity in RJBs, 6 proteins in ribosome pathways related to protein synthesis, MRJP1 (the most abundant protein in RJ), and 3 enzymes associated with energy production: UGP2, MDH, and gltA, were validated by WB analysis (Fig. 6*E*, 6*F*, and 6*G*). Although there were some differences in the abundance levels of RpS26, RpS3A (Fig. 6g), and MRJP1 (Fig. 6*F*) between the proteome data and WB assays, the abundance levels of RpL13, RpL28, RpS4, 60S ribosomal protein L4 (RpL4), UGP2, MDH, and gltA were significantly higher in RJBs than in ITBs in both assays (Fig. 6*F* and 6*G*).

**Fig. 7.**
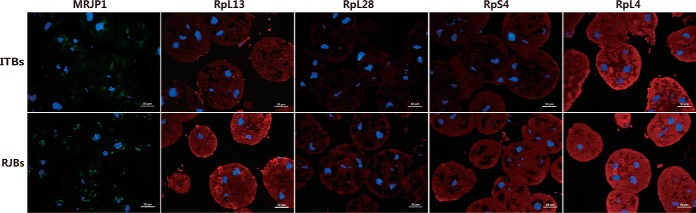
**Images of the immunochemistry staining of proteins MRJP1, RpL13, RpL28, RpS4, and RpL4 in NB the HGs of both Italian bees (ITBs) and high royal jelly producing bees (RJBs).** Blue is nuclei stained by 4′, 6-diamidino-2-phenylindole. The red fluorescence is stained with Cy3-conjugated antibodies. Scale bars represent 50 μm.

To re-confirm the functions of these proteins in enhancing the activity of RJ in RJBs, the expression levels of MRJP1, RpL4, RpS4, RpL13, and RpL28 in NBs of ITBs and RJBs were also visualized by IHC and compared (Fig. 7). The entire glands were dyed with the DAPI. Because of the high abundance of the secretory protein MRJP1 in HGs, a green fluorescence FITC with lower background was used. The intensity and brightness of labeled MRJP1 in RJBs were significantly higher than those in ITBs, consistent with the WB analysis, confirming the enhanced secretory performance of MRJPs in RJBs relative to ITBs. Moreover, the higher expression levels of MRJP1, RpL13, RpL28, and RpS4 were also found by the stronger staining with Cy3 at the secretory cells in HGs of RJB NBs, relative to those of ITBs.

##### Behavioral Transition to Confirm the Contributions of Task-driven Proteins in the Secretion of RJ

**Fig. 8.**
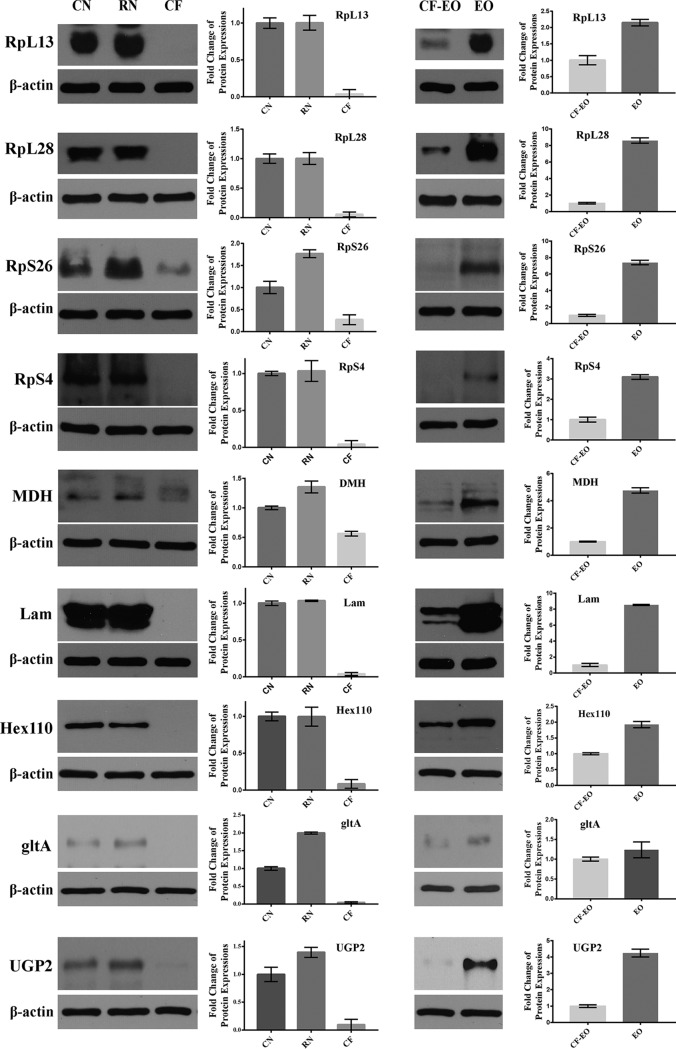
**Verification of protein expressions implicated in protein synthesis and energy metabolism in hypopharyngeal glands dependent on behavior tested by western-blotting assay.** The protein β-actin is used as a reference. CN, normal nurse bees (NBs) as control; RN, reverted NBs; CF, normal forager bees (FBs) as control; CF-EO, normal FBs as control for feeding sugar candy alone; EO, treatment of feeding ethyl oleate (EO) to pro-long the nurse behavior.

In a normal colony, age and labor of worker honeybees are closely related, in which nursing behavior is performed during 6–15 days after eclosion. To eliminate the factor of age-dependence and verify that the function of proteins in HGs is related to labor behavior rather than age, two social manipulation experiments were used to collect RNs and PNs. The expressions of RpL13, RpL28, RpS4, RpS26, MDH, UGP2, gltA, Hex110, and Lam were significantly upregulated in both RNs and EO-induced PNs and were in line with the NBs in a normal colony (Fig. 8). In control FBs, however, the expression levels of these proteins were decreased (Fig. 8). Although the ages of RNs and PNs were the same as those of the regular FBs, the highly abundant above-mentioned proteins and highly induced secretory activity of HGs in both RNs and PNs were positively correlated with RJ secretion and regulated by gland behavioral demands.

## DISCUSSION

To gain novel mechanistic insight into the ontogeny and function of HGs and the enhanced secretory activity of voluminous RJ production in RJBs relative to that in ITBs, the age-specific proteomes of the HGs during adult age in both stocks were investigated and compared. Proteins involved in gland enhanced performance of RJ secretory activity were biochemically and biologically confirmed. An unprecedented depth of HG proteome coverage (2,375 protein groups) was attained, as compared with previous works (9, 50, 53, 56–58). The two bee strains have displayed a similar proteome program to drive their gland development and function by selectively activated functional groups in NEBs, NBs, and FBs to promote gland growth, RJ secretion, and enzyme secretion for brewing honey and pollen, respectively (3, 53, 56). Notably, in the HGs of RJB NBs, a wide spectra of functional groups, particularly in protein synthesis and energy supplement, are induced to prime the enhanced activity for the high performance of RJ secretion relative to ITBs. Furthermore, the stronger secretory activity of HGs in RJB NBs in ribosome pathway and energy metabolism is confirmed further by two social manipulation experiments and further biochemical testing.

### 

#### 

##### Proteome Reveals an Age-specific Strategy To Underpin the Ontogeny and Function of HGs in Both Bee Stocks

Although the RJBs have been genetically selected from ITBs to strengthen the activity of HGs for improved RJ production over decades, the HGs of both stocks carry out age-specific ontogeny and function based on age development. This is manifested by the proteome of both lines in the same stage gathered together in both the PCA and cluster analysis of protein abundance levels. Specifically, the cluster of NEBs far from NBs or FBs in the PCA and the abundant enrichment of functional groups in NEBs relative to NBs or FBs suggest a clear age-based molecular distinction during the HG development. Moreover, the proteomes in parallel stages of HGs in both strains clustered together suggest that the distinct proteome programs have shaped to consolidate the age-specific biological missions.

In NEBs, the HGs are morphologically and physiologically immature and their physiological maturity is crucial to perform their age-dependent tasks (2, 3, 10, 12, 59). To this effect, bulks of protein molecules must be synthesized to promote cell proliferation and tissue development, and intensive biological fuels are involved to maintain this synthesis (9, 50, 56, 60). Compared with NBs and FBs, the exclusively enriched functional groups by upregulated proteins in NEBs in both bee lines related to ribosome biogenesis in eukaryotes, valine, leucine and isoleucine degradation, and proteasome are suggestive of the fact that protein metabolism is vital to stimulate HG development (9, 60). On the other hand, biological fuel supplement is critical for the development of HGs in terms of protein synthesis, cell division, and organ formation (56). The uniquely enriched pathways in NEBs, such as fatty acid degradation and ribonucleoprotein complex biogenesis, indicate that biochemical energy also plays a vital role in driving the young gland growth and tissue construction in HGs (61, 62). Furthermore, Hex 110 is an insect storage protein abundant in the larval fat body and hemolymph to support larval development as an energy source (52, 63, 64). Here, the high abundance level of Hex110 in NEBs validated by WB indicates its role as an element to continually support the energy requirements in HGs for early development and the following strong secretory behavior.

The major role of NBs is specialized in secreting RJ to feed honeybee queens and young larvae (2). This is evidenced by the highly abundant MRJP1 in the HGs of NBs by both WB and IHC assays. Hence, protein synthesis is of paramount importance for RJ production in the HGs of NBs. To this end, the enriched ribosome pathway is a manifestation that protein synthesis machinery is critical for the HGs of NBs, compared with the two other ages. This is in accordance with the distinct proteome of NBs, relative to NEBs and FBs in both stocks, which have been shaped to prime the physiological demand of RJ secretion (9, 57). Furthermore, the upregulated proteins in ribosome pathway in NBs, compared with NEBs and FBs in both bee lines, was validated by WB for RpL4, RpL13, RpL28, RpS4, and RpS3A. This further demonstrates their roles in consolidating the synthesis of protein molecules to accomplish the task of RJ secretion by HGs at this stage.

In NBs, secretory vesicles have developed on day 3, and the volumes of the acini as well as the numbers of secretory vesicles peak around day 6–10. Thereafter, those vesicles are decreased or not visible in the HG cells of FBs about 3 weeks after eclosion (3). During the ontogenic process of HGs, the intermediate filaments are critical for the cytoskeletal component to support the normal shape of HGs that is required for proper functioning in NBs (8). Here, the upregulated fibrous protein Lam (2.87 fold) in NBs is suggestive of its role as both a structural and functional element of the cytoskeleton (8, 65, 66). Moreover, the stronger expression of β-actin, the maturity and larger morphology of the secretory cells of acini in the HGs of NBs, as compared with NEBs and FBs by IHC localization, are in concordance with cytoskeleton structural alteration. Again, the cross sections of actin rings in the IHC image occupied the cytoplasm of the secretory cells of HGs and held the end apparatus in place within the in-pocket of the secretory cell apical surface. Furthermore, the presence of actin-bound vesicular structures alongside the canaliculus during HG development indicates that the acinus surface increase is fed by the lateral fusion of vesicles. This morphological evidence indicates that the HGs of NBs have adapted to behavioral alterations to cement the secretory activity of RJ (8, 56, 65).

Once NBs biologically transform to FBs, the dominant roles of HGs are known to be for collecting nectar and pollen from flowers and to further divert the nectar into honey by adding convertase (10, 12, 67). To achieve the task performance as FBs, the exclusively enriched pathways associated with fructose and mannose metabolism and starch and sucrose metabolism in FBs, relative to NEBs or NBs, are indicative of the fact that carbohydrate metabolism is critical to ensure task performance of HGs during foraging activities (9). The upregulated proteins related to the pathway of starch and sucrose metabolism indicate their induced functions in producing enzymes implicated in converting sucrose into its simple sugar in FBs (11).

##### Stronger Activity of Protein and Energy Supplement Is Critical to Boost the Enhanced Secretory Activity of RJ in HGs of RJB NBs

The specific duties of the NBs are to care for and feed the queen and young larvae with RJ (2, 4, 10, 68, 69). An over 10-fold higher RJ yield and larger acini diameter of HGs in RJBs than in ITBs is solid evidence that the NBs of RJBs have developed a stronger ability in their HGs to produce a large amount of RJ, as compared with ITBs (9, 30, 32). To this effect, a wide cascade of functional groups associated with protein synthesis are exclusively enriched in the HGs of RJB NBs, which signifies their driving force to accomplish an elevated HG function for RJ secretion. For instance, protein export is vital for protein synthesis via transcriptional and translational machinery (70). Additionally, spliceosome is a molecular machine in producing pre-mRNAs by splicing out intronic nucleic acids and then translating them into proteins in ribosomes (71), which are imperative for the regulation of biological processes during regular HG development and RJ secretion (56).

Ribosomal proteins are key components in the translational machinery, vital for synthesis of the proteins used as cellular building blocks for tissue construction and cell development of the HGs (50, 56). To be noted, the upregulated 52 ribosomal proteins in NBs of RJBs relative to ITBs, such as RpS27 (4.07-fold), RpL13 (2.67-fold), RpL4 (2.59-fold), RpS4 (2.59-fold), RpS26 (2.26-fold), RpS3A (2.13-fold), RpL28 (2.09-fold), provide solid evidence that protein biosynthesis in the HGs of RJBs is enhanced to consolidate RJ production. This is because the maintenance of HG function and high secretory performance of RJ demands a bulk of protein material for the functional running of HGs (9). Ribosomal proteins S4, L3, L4, and L13 are involved in rRNA transcription antitermination, which lead to increases in terminator read-through (Martha Torres, 2001). A typical example is S4 playing a regulatory role in both ribosome assembly and rRNA synthesis (72). L28 is critical for protein synthesis in eukaryotes for its enrichment in higher protein synthesis activity (Yang, 2013). The stronger expressions of RpL4, RpL13, RpL28, and RpS4 in NBs of RJBs than of those in ITBs, validated by WB and IHC, indicate that the function of the highly activated ribosome pathway is to meet the demand for large volumes of protein synthesis in NBs of RJBs (9, 56).

Cells and organs receive most of their molecular energy from a long series of reactions that combine oxygen and glucose, forming carbon dioxide and water, thus creating ATP and NADH. The uniquely enriched TCA cycle in NBs of RJBs suggests that energetic fuel is key in facilitating proper cell maintenance during RJ secretion and the physiological functions of HGs in NBs (56). Moreover, the upregulated proteins implicated in energy production, found both in proteome data and WB assay: NADH dehydrogenase (2.6-fold), UGP2 (1.5-fold), gltA (2.9-fold), and MDH (2.33-fold) in HGs of RJBs, relative to in those of ITBs, indicate the high requirement of metabolic energy in providing the cells with readily usable forms of fuels (73–75). For instance, UGP2 plays an important role in the generation of UDP-glucose and is highly active in tissues involved in glycogenesis in higher animals (73, 76, 77). GltA exists as a pace-making enzyme in the first step of the TCA (78). MDH can reversibly catalyze the oxidation of malate to oxaloacetate involved in the TCA (79). These findings are indicative of the fact that the enhanced energy metabolism is the power source of secretory behavior and support the strong secretion performance of RJB HGs. Therefore, the elevated levels of these protein expressions are vital for the secretory performance of HGs.

##### Behavioral Regulation Confirms Key Proteins Enhance High RJ Production

Protein expressions are associated with honeybee behavior regulation (80). The unique plasticity of ontogeny in honeybee workers is a good model to investigate whether protein expression is related to age or labor division in HGs. Here, to distinguish between the possible influence of age-development and behavioral performance, we manually manipulated the social structure of colonies, in which nursing activity was prolonged or foragers were forced to revert to nursing activity. The significantly enhanced expressions of RpL13, RpL28, RpS4, RpS26, MDH, Lam, Hex110, gltA, and UGP2 in reverted NBs and delayed NBs demonstrate that they have stronger relationships with nursing behavior and in turn RJ secretion. These findings are indicative of the fact that ribosomal proteins, rather than age, are key regulators involved in the secretory activity of the HG to boost protein synthesis.

## CONCLUSIONS

The HGs of both bee stocks have similar proteome architectures to cement their HG development and function during adult life to consolidate their specific biological duties. In NEBs, abundant biological processes and pathways are activated for morphological and physiological development of HGs. More powerful activities in protein synthesis, energy metabolism, and cytoskeletal support are vital for RJ production in NBs, and enzyme secretion is specific in FBs. However, the biological functions of HGs involved in protein synthesis and energy metabolism are more strongly enhanced in RJBs, compared with those in ITBs. Moreover, the stronger expression of RpL13, RpS4, RpS26, RpL28, MDH, UGP2, gltA, Hex110, and Lam in regular NBs and behavior manipulated NBs demonstrates their critical roles in enhancing RJ secretory activity in RJB NBs. Future research may consider how different floral resources might affect the morphological and physiological response of the honeybees.

## DATA AVAILABILITY

The LC-MS/MS data have been deposited to the ProteomeXchange Consortium (http://proteomecentral.proteomexchange.org) via the PRIDE partner repository with the data set identifier: PXD010898.

## Supplementary Material

supplemental Fig. S1

Supplemental Figures

Supplemental tables

## Footnotes

^1^ The abbreviations used are:

HG: hypopharyngeal glands
RJ: royal jelly
ITBs: Italian bees
RJBs: high royal jelly producing bees
MGs: mandibular glands
NEBs: newly emerged bees
NBs: nurse bees
FBs: forager bees
WB: western-blotting
RNs: reversed nurse bees
CFs: control forager bees
EO: ethyl oleate
PNs: prolonged nurse bees
IHC: immunohistochemistry
MRJP1: major royal jelly protein 1
RpL: 60S ribosomal protein
RpS: 40S ribosomal protein
Hex110: hexamerin 110
Lam: Lamin Dm0
MDH: Malate dehydrogenase
UGP2: UTP-glucose-1-phosphate uridylyltransferase
gltA: citrate synthase
10-HDA: 10-hydroxy-2-decenoic acid
2-DE: two-dimensional electrophoresis-based
MS: Mass spectrometry
DAPI: 4′,6-diamidino-2-phenylindole
ER: endoplasmic reticulum
PCA: principal component analysis
GO: Gene Ontology
FDR: false discovery rate
QMP: queen mandibular pheromone
DTT: dithiothreitol
TIC: total ion current
DAPI: 4′, 6-diamidino-2-phenylindole.
