# Supplementary material for: In-depth Proteome of the Hypopharyngeal Glands of Honeybee Workers Reveals Highly Activated Protein and Energy Metabolism in Priming the Secretion of Royal Jelly
Source: Mol Cell Proteomics. 2019 Jan 7;18(4):606–21. doi: 10.1074/mcp.RA118.001257 (PMC6442370; doi:10.1074/mcp.RA118.001257)
Supplement: supplemental Fig. S1 [file RA118.001257_index.html]

Supplement to In-depth Proteome of the Hypopharyngeal Glands of Honeybee Workers Reveals Highly Activated Protein and Energy Metabolism in Priming the Secretion of Royal Jelly | Molecular & Cellular Proteomics

## Supplemental Data

- Supplemental Figures - There are five supplemental figures in this file.
- Supplemental tables - 24 Supplemental tables
