# Supplementary material for: In-depth Proteome of the Hypopharyngeal Glands of Honeybee Workers Reveals Highly Activated Protein and Energy Metabolism in Priming the Secretion of Royal Jelly
Source: Mol Cell Proteomics. 2019 Jan 7;18(4):606–21. doi: 10.1074/mcp.RA118.001257 (PMC6442370; doi:10.1074/mcp.RA118.001257)

Fig. S1 Behavioural manipulation experiments. We manage two behavioural manipulation experiments to performance behavior manipulation for sampling the revered nurse bees (NBs) and prolonged NBs. See Experimental procedures section for details. FBs, forager bees.


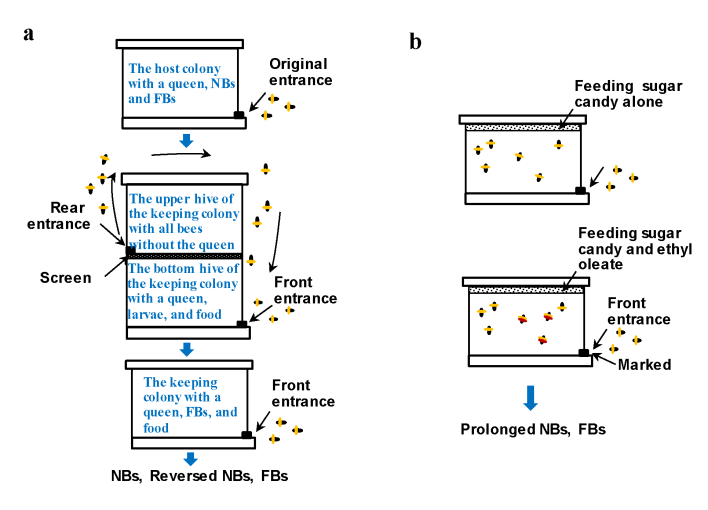


Fig. S2 The qualitative comparisons of time-resolved proteome in hypopharyngeal glands (HGs) from newly emerged bees (NEBs), nursing bees (NBs), and forager bees (FBs) in Italian honeybees. The identified proteins in NEBs, NBs, and FBs are analyzed by ClueGO to compare the functional classes and pathways specifically enriched by three data sets.

a, Pie chart overview of the significantly enriched common functional classes and pathways shared by NEBs, NBs, and FBs;

b and c, The unique functional classes and pathways significantly enriched by NEBs;

d and e, The unique functional classes and pathways significantly enriched by NBs;

f, and g, The unique functional classes and pathways significantly enriched by FBs;

The details of the enrichment analysis results see and Table S7.

% genes/Term stands for the proportion of genes enriched in corresponding functional groups. The bars with the same color present they belong to the same functional groups. The numbers strand for the genes enriched to the corresponding functional groups. *, *P* < 0.05; **, *P* < 0.01.


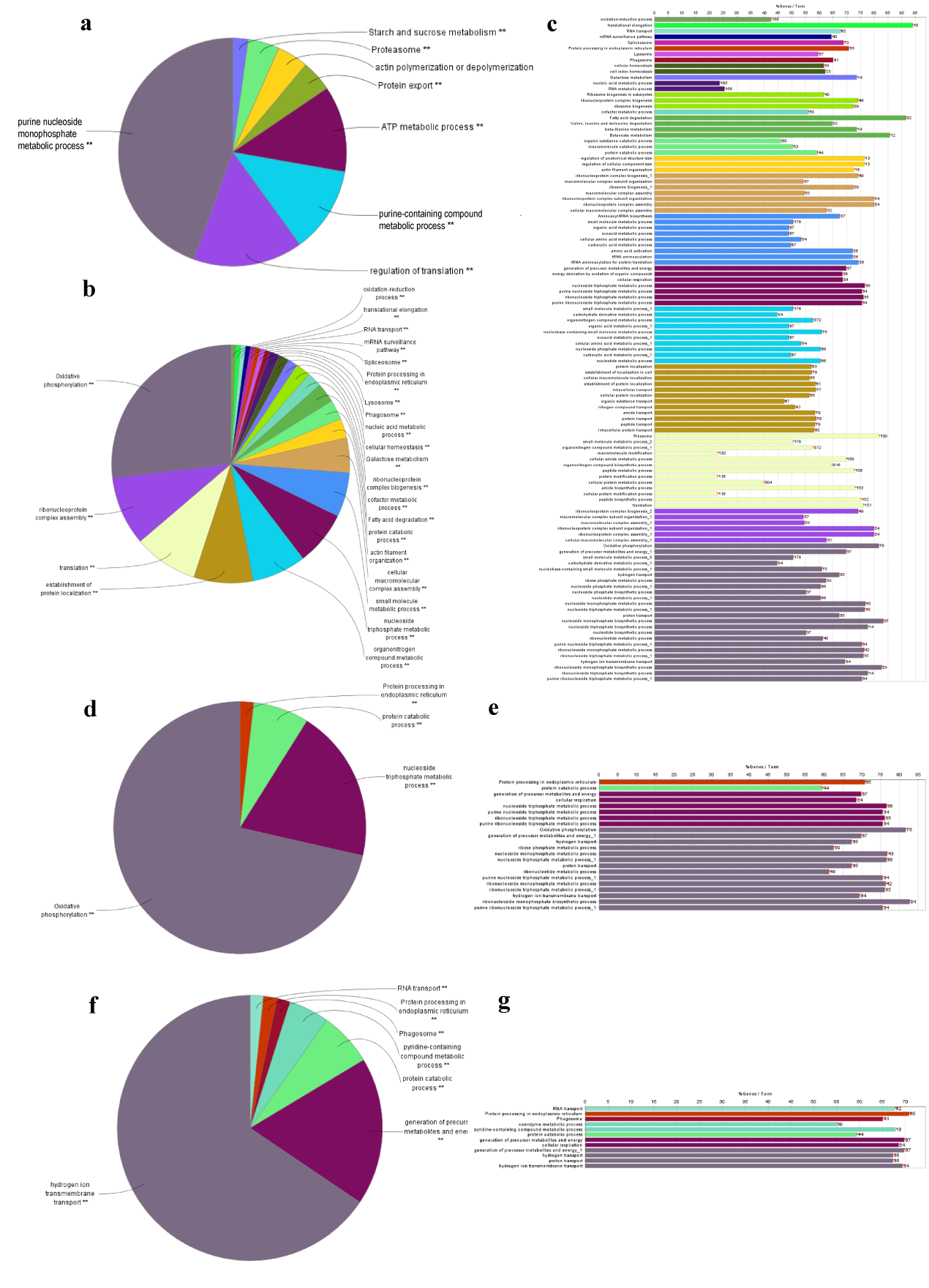


Fig. S3 The qualitative comparisons of time-resolved proteome in hypopharyngeal glands from newly emerged bees (NEBs), nursing bees (NBs), and forager bees (FBs) in high royal jelly honeybees. The identified protein in NEBs, NBs, and FBs are analyzed by ClueGO to compare the functional classes and pathways specifically enriched by three data sets.

a, Pie chart overview of the significantly enriched common functional classes and pathways shared by NEBs, NBs, and FBs;

b and c, The unique functional classes and pathways significantly enriched by NEBs;

d and e, The unique functional classes and pathways significantly enriched by NBs;

f, and g, The unique functional classes and pathways significantly enriched by FBs;

The details of the enrichment analysis results see and Table S11.

% genes/Term stands for the proportion of genes enriched in corresponding functional groups. The bars with the same color present they belong to the same functional groups. The numbers strand for the genes enriched to the corresponding functional groups. *, *P* < 0.05; **, *P* < 0.01.


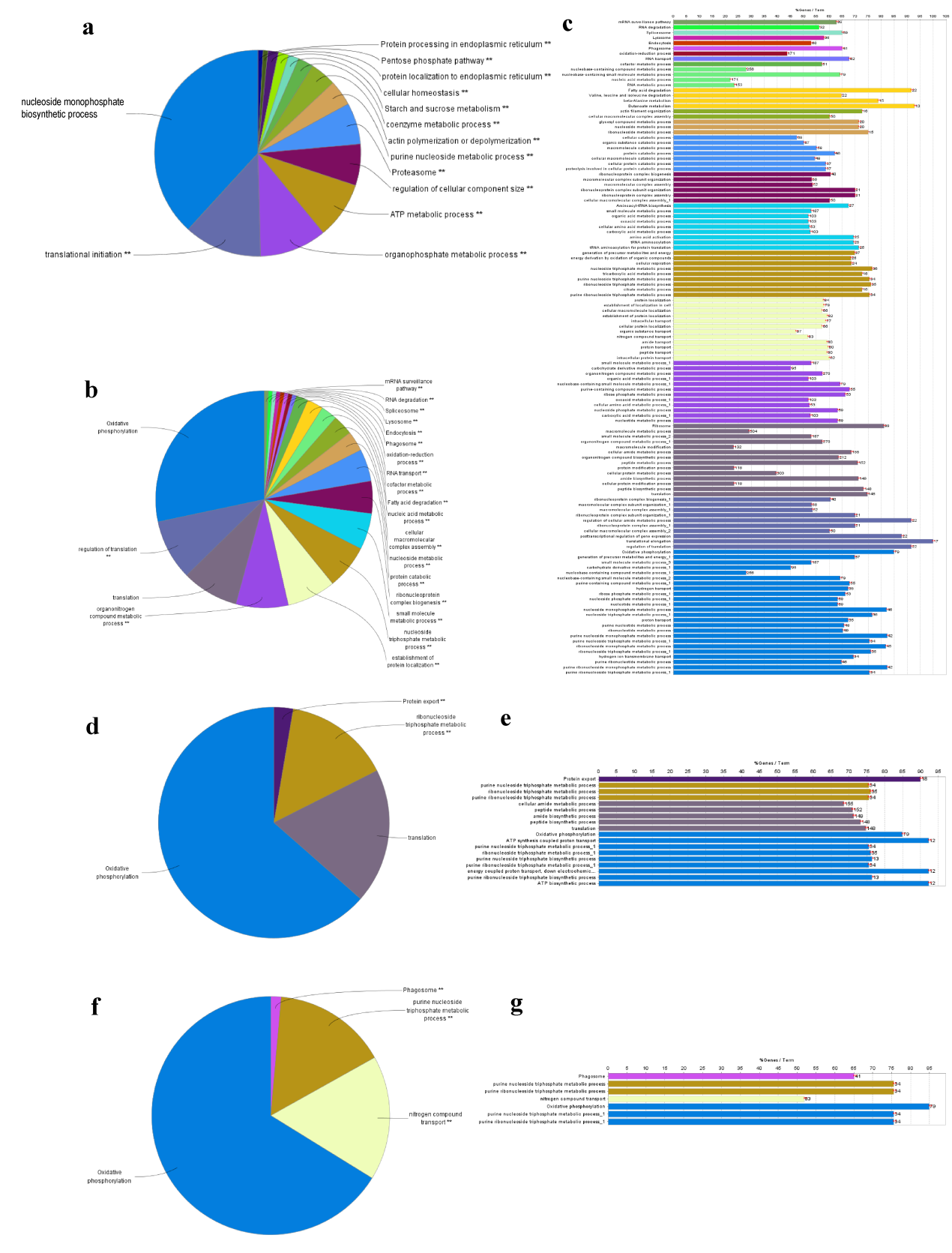


**Fig. S4 Comparisons of hypopharyngeal gland (HGs) proteins in newly emerged bees (NEBs) between Italian bees (ITBs) and royal jelly bees (RJBs).** a, b, and c, Qualitative comparison of identified HG proteins in NEBs between ITBs and RJBs using ClueGO; The protein identified in ITBs and RJBs are analyzed by ClueGO to compare the functional classes and pathways specifically enriched by two data sets. a, Pie chart overview of the significantly enriched functional classes and pathways shared by both ITB and RJB NEBs; b and c, The unique functional classes and pathways enriched by NEBs of ITBs and RJBs, respectively. The details of the enrichment analysis results see Table S16.

d and e, Quantitative comparison of HG proteins of NEBs between RJBs and ITBs using ClueGO; d, The significantly enriched functional classes and pathways by up-regulated proteins in NEBs of ITBs compared to RJBs (fold change ≥ 2 and *P* < 0.05); e, The significantly enriched functional classes and pathways by up-regulated proteins in NEBs of RJBs relative to ITBs (fold change ≥ 2 and *P* < 0.05). The details of the enrichment analysis results see Table S18.

% genes/Term stands for the proportion of genes enriched in corresponding functional groups. The bars with the same color denote the same functional groups they are belonged to. The numbers strand for the genes enriched to the corresponding functional groups.

f, The difference expressions of proteins MRJP1, RpL28, and Hex110 in NEBs between ITBs and RJBs tested by western-blotting assay. Protein β-actin is used as a loading control. IT1, NEBs of ITBs; RJ1, NEBs of RJBs.

g, The relative abundance level of protein MRJP1, RpL28, and Hex110; upper panel, the relative fold changes of the protein expressions of RJBs compared to ITBs tested by WB; lower panel, the fold changes of protein expressions calculated by label free quantification.

*, *P* < 0.05; **, *P* < 0.01.


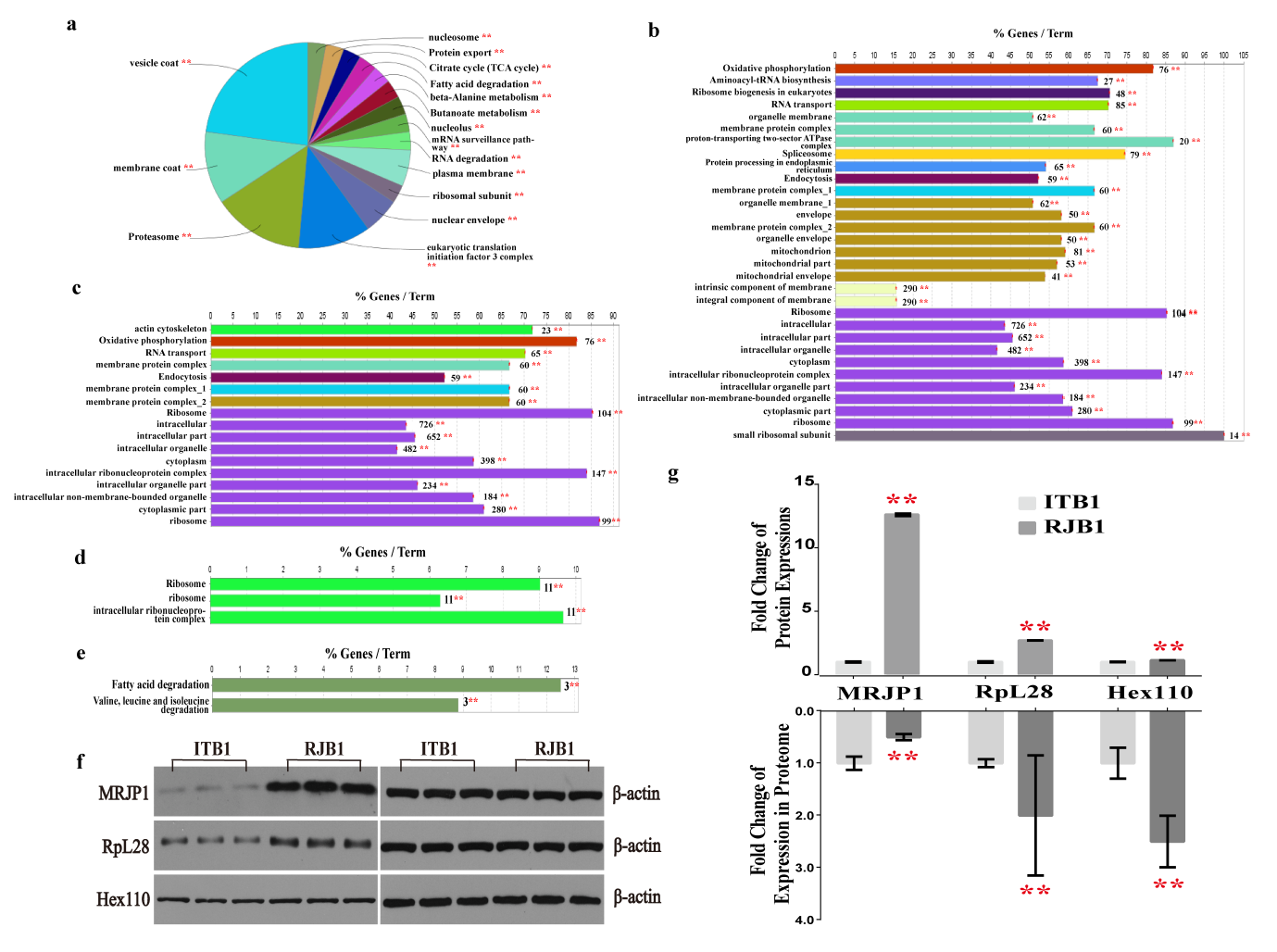


**Fig. S5 Comparisons of hypopharyngeal gland (HG) proteins of forager bees (FBs) between Italian bees (ITBs) and royal jelly bees (RJBs).** a, b, and c, Qualitative comparison of identified HG proteins of FBs between ITBs and RJBs using ClueGO; The protein identified in ITBs and RJBs are analyzed by ClueGO to compare the functional classes and pathways specifically enriched by two data sets. a, Pie chart overview of the significantly enriched common functional classes and pathways shared by both ITB and RJB FBs; b and c, The unique functional classes and pathways significantly enriched by FBs of ITBs and RJBs, respectively. The details of the enrichment analysis results see Table S22.

d and e, Quantitative comparison of HG proteins of FBs between ITBs and RJBs using ClueGO; d, The significantly enriched functional classes and pathways by up-regulated proteins in FBs of ITBs compared to RJBs (fold change ≥ 2 and *P* < 0.05); e, The significantly enriched functional classes and pathways by up-regulated proteins in FBs of RJBs relative to ITBs (fold change ≥ 2 and *P* < 0.05). The details of the enrichment analysis results see Table S24.

% genes/Term stands for the proportion of genes enriched in corresponding functional groups. The bars with the same color present they belong to the same functional groups. The numbers strand for the genes enriched to the corresponding functional groups.

f, The difference expressions of proteins MRJP1 in FBs between ITBs and RJBs tested by western-blotting assay. Protein β-actin is used as a loading control. ITF, FBs of ITBs; RJF, FBs of RJBs.

g, The relative expression value of protein MRJP1; left, the relative fold changes of MRJP1 expression of RJBs compared to ITBs tested by WB; right, the fold changes of MRJP1 calculated by label free quantification.

*, *P* < 0.05; **, *P* < 0.01.


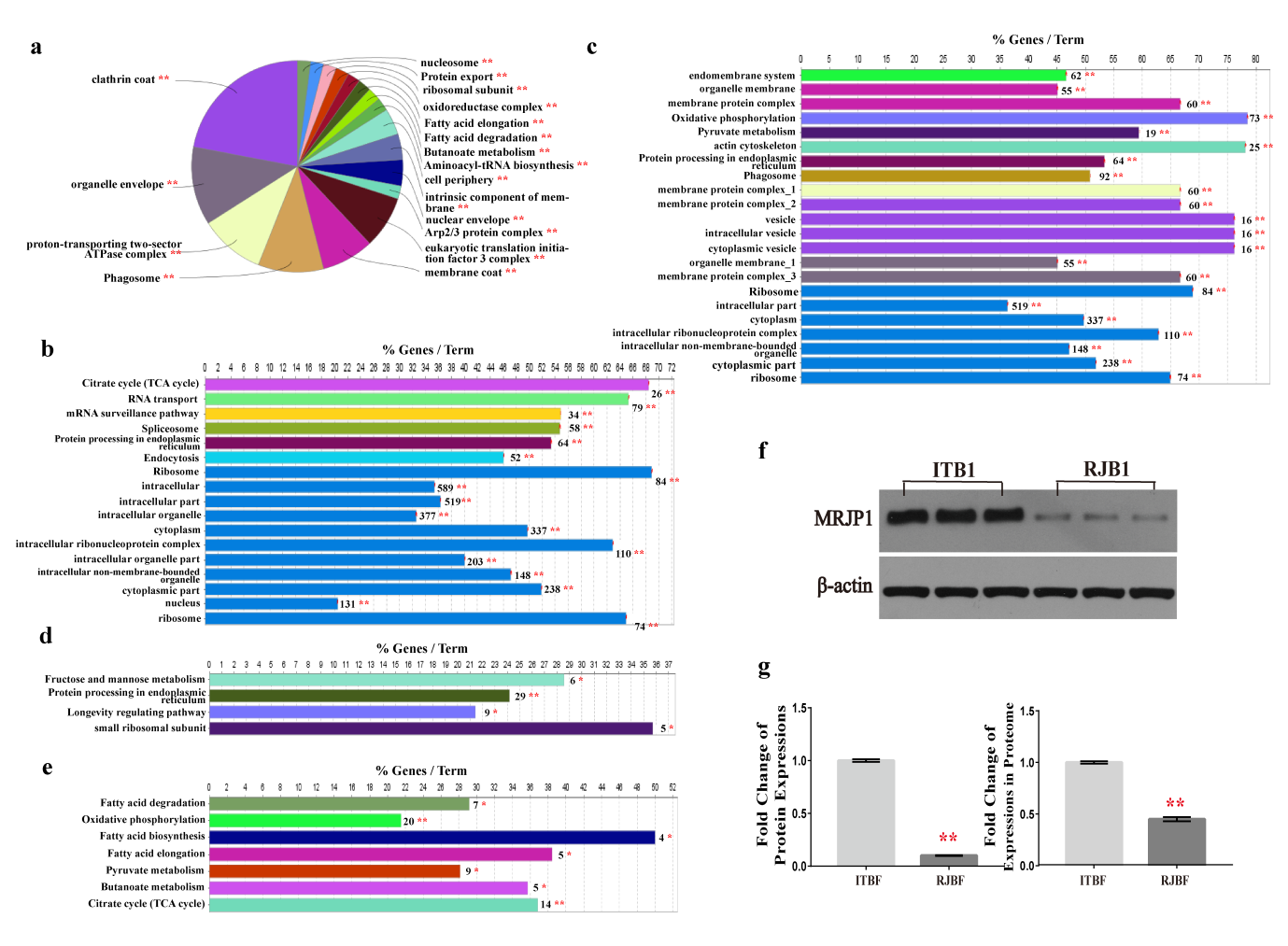

Supplement: Supplemental Figures [file 142358_0_supp_245110_pj7d1m.docx]
